# The extent of myocardium at risk for LAD, RCA and LCx using contrast enhanced SSFP and T2-weighted imaging

**DOI:** 10.1186/1532-429X-17-S1-P139

**Published:** 2015-02-03

**Authors:** David Nordlund, Einar Heiberg, Marcus Carlsson, Ernst-Torben Frûnd, Pavel Hoffmann, Jan Erik Nordrehaug, Sasha Koul, Dan Atar, David Erlinge, Henrik Engblom, Håkan Arheden

**Affiliations:** Cardiac MR group Lund, Dept. of Clinical Physiology, Lund University, Lund, Sweden; Dept. of Radiology, Odense University Hospital, Odense, Denmark; Dept. of Cardiology B, Oslo University Hospital Ullevål, and Faculty of Medicine, University of Oslo, Oslo, Norway; Dept. of Cardiology, Haukeland University hospital, Bergen, Norway; Dept. of Cardiology, Lund University, Lund, Sweden

## Background

Contrast enhanced SSFP (CE-SSFP) and T2-weighted triple inversion recovery imaging (T2w) have both been clinically validated for determining myocardium at risk (MaR) by cardiovascular magnetic resonance (CMR), using myocardial perfusion SPECT (MPS) as reference standard. Previously, MPS has been used to describe the coronary perfusion territories during myocardial ischemia. Compared to MPS, CMR offers superior image quality and logistical advantages. The aim of this study was to describe the coronary perfusion territories of LAD, RCA and LCx based on CMR data using CE-SSFP and T2w in patients after ST-elevation myocardial infarction.

## Methods

CE-SSFP and T2w data from the recently published international multi-center trials CHILL-MI and MITOCARE, was used to assess MaR. CE-SSFP images from 206/212 patients (6 excluded due to inability to detect MaR) and T2w images from 147/212 patients (12 excluded due to missing data, 53 due to inability to detect MaR) were included. Late gadolinium enhancement (LGE) imaging was used to assess infarct size. Imaging was performed on systems from three different vendors (Siemens, Philips, GE) and data was analyzed by a core laboratory. Culprit vessel was determined using angiography.

## Results

Average size of MaR was greater for LAD (CE-SSFP: 44±10%, T2w: 44±9% of the left ventricle [LV], p-value < 0.0001) than for both LCx (CE-SSFP: 30±9%, T2w: 30±12% of the LV) and RCA (CE-SSFP: 31±7%, T2w: 30±8% of the LV). A comparison of coronary perfusion territories between CMR and previously published MPS data is shown in 1. All patients had a smaller infarct by LGE compared to MaR by CE-SSFP and all patients but one by T2w (2).

**Figure 1:**
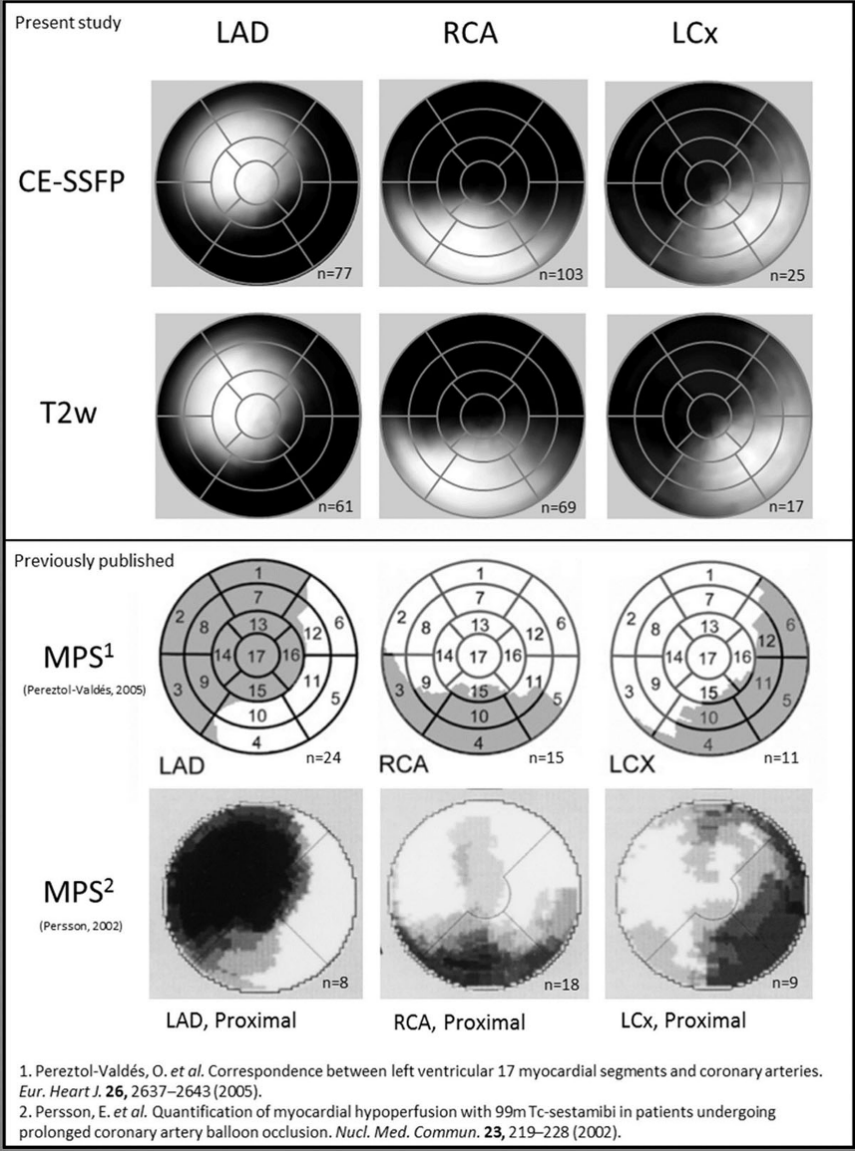
**Polar plot representation of coronary perfusion territories.** The top and second rows show CE-SSFP and T2w images from the present study. The third row shows maximal extent of perfusion territories using MPS (Pereztol-Valdés^1^) and the bottom row shows perfusion territories also using MPS (Persson^2^). The left column shows LAD, the central column RCA and the right column LCx. The intensity of the plot is proportional to the fraction of patients with MaR in that area.

**Figure 2:**
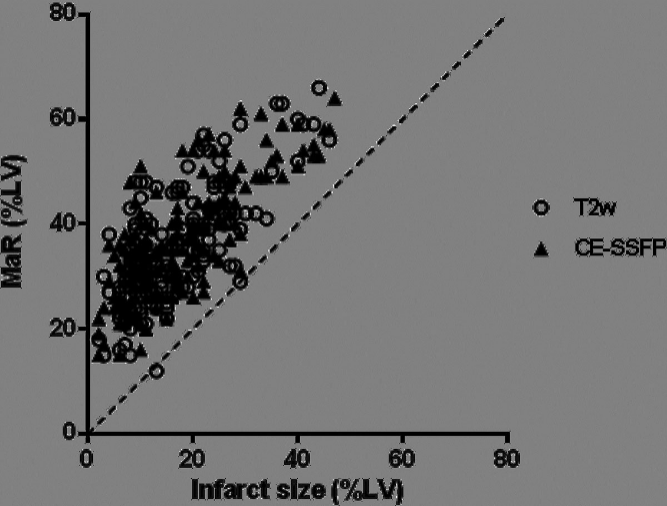
MaR by T2w and CE-SSFP on the Y-axis vs Infarct size on the x-axis (both expressed as % of LV volume).

## Conclusions

The perfusion territories of the three main coronary arteries were described using CE-SSFP and T2w in patients with acute myocardial infarction and resemble previous territories by MPS, with expected overlap between RCA and LCx.
